# Supplementary material for: SyNDI: synchronous network data integration framework
Source: BMC Bioinformatics. 2018 Nov 6;19:403. doi: 10.1186/s12859-018-2426-5 (PMC6219086; doi:10.1186/s12859-018-2426-5)
Supplement: Supplementary file 5 — Files of the example presented in Additional file 4. This additional file is a zip package that contains all files used in Additional file 4. Detailed descriptions of each of these files are presented in a README file included in this zip package. (GZ 30411 kb) [file 12859_2018_2426_MOESM5_ESM.gz › SyNDITools/Meme2Fimo/meme_4.9.0_4.tar.gz!/meme_4.9.0/website/cgi-bin/tomtom.tmpl]

Tomtom - Submission form


You may use both IUPAC codes and
regular expression bracket expressions. For example "NTRGGTCAN" and
"[ACGT]T[AG]GGTCA[ACGT]" are equivalent approximations of the example
query shown on the right.

[ close ]

A motif is generated from the specified IUPAC sequence fragment by
distributing the site counts equally over each possible residue. The
pseudocount is added to each residue in proportion to the background.
Thus the total count for a position is the site count plus the
pseudocount.

For example suppose one of the IUPAC codes entered is 'R'. The IUPAC
code 'R' means that both Adenine and Guanine are possible, so with a site
count of 10, pseudocount of 1 and a uniform background; the probability
of Adenine and Guanine would be:  

while the probility of Cytosine and Thymine would be:

[ close ]

The background is used to determine how the pseudocount is distributed.
While it is conventional to have the background sum to one these fields
will be normalised so you don't have to be exact. You may even use counts
instead.

[ close ]

A matrix is rectangular grid of numbers which shows the relative
frequency a nucleotide will occur at a specific position. Two orientations
are possible. For example:

|  |  |
| --- | --- |
| Row Matrix | Column Matrix |||  |  |
| --- | --- |
| \| A \| C \| G \| T; 1 \| 3 \| 8 \| 14 \| 3; 2 \| 3 \| 0 \| 0 \| 25; 3 \| 19 \| 0 \| 9 \| 0; 4 \| 0 \| 1 \| 27 \| 0; 5 \| 1 \| 0 \| 26 \| 1; 6 \| 0 \| 1 \| 4 \| 23; 7 \| 2 \| 23 \| 3 \| 0; 8 \| 26 \| 1 \| 0 \| 1; 9 \| 5 \| 15 \| 4 \| 4 | \| 1 \| 2 \| 3 \| 4 \| 5 \| 6 \| 7 \| 8 \| 9; A \| 3 \| 3 \| 19 \| 0 \| 1 \| 0 \| 2 \| 26 \| 5; C \| 8 \| 0 \| 0 \| 1 \| 0 \| 1 \| 23 \| 1 \| 15; G \| 14 \| 0 \| 9 \| 27 \| 26 \| 4 \| 3 \| 0 \| 4; T \| 3 \| 25 \| 0 \| 0 \| 1 \| 23 \| 0 \| 1 \| 4 |
| Click an example to use it as the input. |

Tomtom automatically detects the orientation but if for some reason the
correct orientation isn't being detected it can be specified in the
advanced section.

[ close ]

A matrix is column oriented if it has 4 rows and any number of columns.
The original Tomtom web format was column oriented. A matrix is row
oriented if it has 4 columns and any number of rows.

[ close ]

The site count is only used when a probability matrix is specified,
otherwise it is derived from the matrix.

The pseudocount is added to each residue in proportion to the
background. Thus the total count for a position is the site count plus
the pseudocount.

[ close ]

By default only the first motif is searched but this can be changed in
the advanced section. To search with more than one motif Tomtom must
process your request as part of a queue and hence a contact email will be
required. See more information on the meme format and the sample MEME DNA motifs.

[ close ]

The filter supports selecting motifs from the file by their name and/or
their order in the file. Any numbers are assumed to refer to the position of
the motif in the file. Any other entry is assumed to be a motif name (or
ID). Motif names can not start with a dash and can only contain
alphanumeric characters plus **:** **\_** **.** and **-**. The
motifs should be separated by spaces.

[ close ]

An uploaded database is a normal MEME DNA motif file with lots of motifs. The uploaded database must
be smaller than 5MB.

[ close ]

The email is used to notify you of your results but is not stored.

[ close ]

The job description will be included in the notification email you
receive.

[ close ]

Motifs are compared column by column. In the following equations
***X*** is the set of probabilities from one column and
***Y*** the probabilities from the compared column. The subscript
***a*** refers to the letter from the nucleotide alphabet
***A***.

Pearson correlation coefficient


Euclidean distance


Sandelin-Wasserman similarity

[
close ]

There are two ways of specifying the threshold.

*E-value*
:   The expected number of false positives. This may be any positive
    number.

*q-value*
:   Minimum **F**alse **D**iscovery **R**ate at which a result
    is declared significant. This is a number between zero and one
    non-inclusive.

[
close ]

Complete scoring takes into account unaligned columns in the
calculation of scores. It is recommended that you leave this feature
enabled unless you are trying to recreate old results.

[
close ]

The background is used to determine how the pseudocount is distributed.
While it is conventional to have the background sum to one these fields
will be normalised so you don't have to be exact. You may even use counts
instead.

[
close ]

Tomtom can search databases installed on the server or search a user
uploaded database in MEME DNA motif
format by selecting the "Upload Your Own Database" option. Uploaded
databases are searched in a queue and so require an email address. For
more information browse the supported databases.

[
close ]

|  |  |
| --- | --- |
|  | MEME Suite  The MEME Suite web application requires the use of JavaScript  Javascript doesn't seem to be available on your browser.  Version  Use this form to submit one or more DNA motifs to Tomtom for comparison against a database of known motifs (e.g., JASPAR or Transfac). Tomtom will rank the motifs in the target database by the *E*-value of the similarity score and produce scoring details and a logo alignment for each significant match.  Data Submission Form Search one or more DNA motifs against a DNA motif database.  Example Output  Preview  Empty sequence   Preview Error Log   Choose the type of motif  checked> IUPAC  Matrix  MEME   Submitted    Input the motif  Enter a IUPAC DNA motif.    Advanced Options  Advanced Options  Enter a site count and pseudocount. sites:      pseudocount:    Enter the background model. A:     C:     G:     T:  Enter a DNA count/probability matrix.    Advanced Options  Advanced Options  Select the orientation. orientation:   autodetect row column   Enter a site count and pseudocount. sites:      pseudocount:    Enter the background model. A:     C:     G:     T:  Select a MEME DNA motif file.   Advanced Options  Advanced Options  Specify which motif(s) to search with.  or  all motifs.   Tomtom will use the submitted motifs.   Choose the motif database to search  Upload Your Own Database  Input the database  Select a MEME motif database file.  Input job queue details  Enter your email address.  Re-enter your email address.   Optionally enter a job description.  Advanced Options  Advanced Options  Select the motif column comparison function  Pearson correlation coefficient Euclidean distance Sandelin-Wasserman similarity  Set the significance threshold  *E*-value *q*-value <  Activate complete scoring  complete scoring |
